# A Prospective Study of Stereotactic Body Radiotherapy (SBRT) with Concomitant Whole-Pelvic Radiotherapy (WPRT) for High-Risk Localized Prostate Cancer Patients Using 1.5 Tesla Magnetic Resonance Guidance: The Preliminary Clinical Outcome

**DOI:** 10.3390/cancers14143484

**Published:** 2022-07-18

**Authors:** Darren M. C. Poon, Jing Yuan, Bin Yang, Oi-Lei Wong, Sin-Ting Chiu, George Chiu, Kin-Yin Cheung, Siu-Ki Yu, Raymond W. H. Yung

**Affiliations:** 1 Comprehensive Oncology Centre, Hong Kong Sanatorium & Hospital, Happy Valley, Hong Kong SAR, China; 2 Research Department, Hong Kong Sanatorium & Hospital, Happy Valley, Hong Kong SAR, China; jyuanbwh@gmail.com (J.Y.); oilei.ol.wong@hksh.com (O.-L.W.); raymondyung@hksh.com (R.W.H.Y.); 3 Medical Physics Department, Hong Kong Sanatorium & Hospital, Happy Valley, Hong Kong SAR, China; kimi.b.yang@hksh.com (B.Y.); kycheung@hksh.com (K.-Y.C.); benyu@hksh.com (S.-K.Y.); 4 Department of Radiotherapy, Hong Kong Sanatorium & Hospital, Happy Valley, Hong Kong SAR, China; sinting.chiu@hksh.com (S.-T.C.); georgechiu@hksh.com (G.C.)

**Keywords:** magnetic resonance-guided stereotactic body radiation therapy (MRgSBRT), high-risk prostate cancer (HR-PC), whole-pelvic radiotherapy (WPRT), toxicity, quality of life (QoL)

## Abstract

**Simple Summary:**

Magnetic resonance (MR)-guided stereotactic body radiotherapy (MRgSBRT) with concomitant whole-pelvic nodal radiotherapy (WPRT) represents a novel radiotherapy paradigm for high-risk prostate cancer (HR-PC), potentially improving online image guidance and clinical outcomes. This is the first prospective study aims to report the preliminary clinical experiences and treatment outcome of 1.5 Tesla adaptive MRgSBRT with concomitant WPRT in HR-PC patients (72.5 ± 6.8 years). Forty-two consecutive localized HR-PC patients were treated by online adaptive MRgSBRT (8 Gy(prostate)/5 Gy(WPRT) × 5 fractions) combined with androgen deprivation therapy (ADT) and followed up (median: 251 days, range: 20–609 days). The maximum cumulative acute gastrointestinal (GI)/ genitourinary (GU) grade 1 and 2 toxicity rates were 19.0%/81.0% and 2.4%/7.1%, respectively. The subacute (>30 days) GI/GU grade 1 and 2 toxicity rates were 21.4%/64.3% and 2.4%/2.4%, respectively. No grade 3 toxicities were reported. Patient-reported quality of life (QoL) showed insignificant changes in urinary, bowel, sexual, and hormonal domain scores during the follow-up period. All patients had early post-MRgSBRT biochemical responses, while biochemical recurrence (prostate-specific antigen nadir + 2 ng/mL) occurred in one patient at month 18. The early results suggested favorable treatment-related toxicities and encouraging patient-reported QoLs, but long-term follow-up is still needed.

**Abstract:**

**Background:** Conventionally fractionated whole-pelvic nodal radiotherapy (WPRT) improves clinical outcome compared to prostate-only RT in high-risk prostate cancer (HR-PC). MR-guided stereotactic body radiotherapy (MRgSBRT) with concomitant WPRT represents a novel radiotherapy (RT) paradigm for HR-PC, potentially improving online image guidance and clinical outcomes. This study aims to report the preliminary clinical experiences and treatment outcome of 1.5 Tesla adaptive MRgSBRT with concomitant WPRT in HR-PC patients. **Materials and methods:** Forty-two consecutive HR-PC patients (72.5 ± 6.8 years) were prospectively enrolled, treated by online adaptive MRgSBRT (8 Gy(prostate)/5 Gy(WPRT) × 5 fractions) combined with androgen deprivation therapy (ADT) and followed up (median: 251 days, range: 20–609 days). Clinical outcomes were measured by gastrointestinal (GI) and genitourinary (GU) toxicities according to the Common Terminology Criteria for Adverse Events (CTCAE) Scale v. 5.0, patient-reported quality of life (QoL) with EPIC (Expanded Prostate Cancer Index Composite) questionnaire, and prostate-specific antigen (PSA) responses. **Results:** All MRgSBRT fractions achieved planning objectives and dose specifications of the targets and organs at risk, and they were successfully delivered. The maximum cumulative acute GI/GU grade 1 and 2 toxicity rates were 19.0%/81.0% and 2.4%/7.1%, respectively. The subacute (>30 days) GI/GU grade 1 and 2 toxicity rates were 21.4%/64.3% and 2.4%/2.4%, respectively. No grade 3 toxicities were reported. QoL showed insignificant changes in urinary, bowel, sexual, and hormonal domain scores during the follow-up period. All patients had early post-MRgSBRT biochemical responses, while biochemical recurrence (PSA nadir + 2 ng/mL) occurred in one patient at month 18. **Conclusions:** To our knowledge, this is the first prospective study that showed the clinical outcomes of MRgSBRT with concomitant WPRT in HR-PC patients. The early results suggested favorable treatment-related toxicities and encouraging patient-reported QoLs, but long-term follow-up is needed to confirm our early results.

## 1. Introduction

Prostate cancer (PC) is the second most common type of cancer in men. There were 1,276,106 new PC cases and 358,989 attributable deaths worldwide in 2018, accounting for ~3.8% of all deaths caused in men in 2018 [1]. Among the newly diagnosed PC cases in the United States, approximately 17–31% of them present with nonmetastatic high-risk (HR) localized PC [2]. Despite the different PC risk stratification algorithms [3,4,5,6,7,8], HR-PC patients have a more than threefold higher risk of death from cancer-related causes compared to low-risk (LR) PC patients [9,10].

Radiotherapy (RT) is a well-established and standard treatment option for PC with all risk levels. To date, hypofractionationed RT is regarded as the preferred fractionation for low-, intermediate-, and high-risk localized PC [11,12,13]. For HR-PC, the combination of androgen deprivation therapy (ADT) and RT has been shown to improve overall survival [14,15,16,17]. Several clinical studies also demonstrated a superior biochemical control in HR-PC by using dose-escalated external beam RT (EBRT) over standard-dose EBRT [18,19,20].

In recent years, the clinical use of stereotactic body radiotherapy (SBRT), a highly conformal RT technique with a dose per fraction of 5.0 Gray (Gy) or higher, has also been actively explored in localized PC RT [21,22,23,24,25]. With compelling clinical evidence, SBRT is currently considered to be an alternative to conventionally fractionated RT for LR-PC and intermediate-risk PC (IR-PC) treatment in the clinical guideline [26]. In contrast, for HR-PC, the role of SBRT remains undetermined due to the paucity of clinical data [24,27,28,29,30]. Only one phase 3 clinical trial of HYPO-RT-PC demonstrated that SBRT delivered with 42.7 Gy in seven fractions (3 days per week) was noninferior to conventionally fractionated RT for IR-PC and HR-PC patients regarding failure-free survival [24]. The long-term patient-reported quality-of-life (QOL) up to 6 years in the HYPO-RT-PC study showed no significant difference in the incidence of clinically relevant deterioration between the SBRT and standard RT groups [23]. Nonetheless, the HR-PC patients only accounted for 11% of total patients (64 and 62 patients in two arms in total) in the HYPO-RT-PC trial, and they did not receive concurrent ADT [8].

One important question in the management of nonmetastatic HR-PC is whether whole-pelvic RT (WPRT) plus prostate-only RT is necessary and/or feasible [31,32,33]. The survival may potentially be enhanced with WPRT as the result of eradication of the pelvic nodal micrometastasis. The definitive role of WPRT has been explored in two randomized studies; however, a robust conclusion in HR-PC is yet to be drawn [34,35]. Until recently, the clinical outcomes of the phase III randomized controlled POP-RT trial showed that WPRT for HR-PC in the conventional fractionation arm improved failure-free survival (BFFS) and disease-free survival (DFS), and it achieved similar overall survival (OS) to the prostate-only RT arm [36]. While awaiting the long-term outcome, WPRT should be routinely considered in the radiotherapy paradigm in management of HR-PC patients [34,35,37].

SBRT with concomitant WPRT represents a novel RT treatment paradigm for HR-PC. It has economical effectiveness with a shorter treatment course and a radiobiologic advantage in delivering a higher biological dose to the prostate primary and pelvic lymph nodes. As in the subset of LR- or IR-PC, SBRT might potentially lead to similar or improved oncological outcomes to conventionally fractionated RT. Few studies [27,38,39,40] have been conducted to explore the feasibility and safety of this novel approach. Most of these studies [27,38,39] preliminarily showed good early tolerance and low toxicity except for the phase I FASTR study [40]. Higher-than-anticipated late toxicities were observed in the phase I FASTR study, and it was terminated before the phase 2 accrual. While the long-term outcome is yet to be reported, caution should be given in implementing SBRT in HR-PC with extended radiotherapy volume, especially if contemporary image guidance is not available.

To date, the reported studies investigating SBRT with concomitant WPRT in HR-PC patients utilized X-ray-based image guidance. The prostate per se, as well as the dominant intraprostatic lesions (DILs), could not be adequately visualized; therefore, invasive fiducial marker implantation was applied, despite magnetic resonance imaging (MRI) often being used in the planning process. Magnetic resonance-guided radiotherapy (MRgRT) [41,42,43,44,45] offers an optimal and novel platform to deliver SBRT with concomitant WPRT in HR-PC and overcome the limitation in the conventional X-ray-based imaging guidance by taking great advantage of the remarkable soft-tissue contrast of MR images. The better visualization of on-the-day anatomies by daily MR images at each fraction also facilitates online plan adaptation without fiducial markers to achieve superior targets coverage and sparing organs at risk (OARs) [46]. A recent simulation study suggested that online adapted MRgRT may reduce the dose to organs at risk (OARs) in HR-PC patients due to the reduced PTV margins derived from inter-fractional daily MRI, which potentially translates to toxicity reduction [47]. However, to date, the reports of clinical outcomes of MRgSBRT with concomitant WPRT in HR-PC remain sparse.

Herein, the major purpose of this study is to report the initial clinical experiences and preliminary treatment outcomes of MR-guided SBRT (MRgSBRT) with WPRT concurrently delivered to HR-PC patients on a 1.5 Tesla MR-LINAC (Unity, Elekta, Stockholm, Sweden) in a cancer institution.

## 2. Material and Methods

### 2.1. Patient Selection

This prospective study was approved by the institutional research ethics committee (REC-2019-09), and patients’ written consent was obtained. Patient inclusion criteria were as follows: age > 18 years; biopsy-proven localized or locally advanced high-risk prostate cancer defined by D’Amico criteria [10] without lymph nodal or distant metastases as diagnosed on recent multiparametric MRI and/or prostate-specific membrane antigen (PSMA) positron emission tomography (PET) imaging; no MRI contraindication. Exclusion criteria were as follows: MRI contraindications; biopsy-proven localized PC with low or intermediate risk defined by D’Amico criteria; previous prostate surgeries or irradiation; previous history of other cancers.

### 2.2. Simulation Scan 

All patients received CT and subsequent MRI simulation scans on the same day with full bladder control, both in the MRgSBRT treatment position. A fiducial marker was unnecessary in MRgRT and, therefore, was not implanted in any patients. Rectal balloons (QLRAD, Miami, FL, USA) with 50–90 mL of saline inflation used for rectal control. MRI simulation was performed on a 1.5 T MR-simulator (Ingenia MR-RT, Philips Healthcare, Best, the Netherlands) using a 3D T2-weighted turbo-spin-echo (3D-T2W-TSE) sequence [48]. For the patients who opted for rectal spacer placement, ~10 mL of the spacer (Barrigel, Palette Life Sciences, Stockholm, Sweden or equivalent) was injected into the perirectal space posterior to rectoprostatic fascia to increase the distance between the prostate and rectum at least 10 days before simulation scan.

### 2.3. Treatment Planning

Tissue contouring was conducted using MIM v.6.9.3 (MIM Software Inc. Cleveland, OH, USA). The clinical target volume (CTV) was contoured by a radiation oncologist on the CT-registered planning MRI, and then propagated to the planning CT images. The CTV consisted of the prostate gland plus the base of the seminal vesicles (SVs). For cT3b cases, the entire seminal vesicles were included in the CTV. Dominant intraprostatic lesions (DILs) were also delineated on the planning MRI by referring to the most recent multiparametric diagnostic and planning MRI, mainly based on T2-weighted MRI and diffusion-weighted imaging (DWI). Pelvic nodal CTV (CTV_L) was delineated starting at the L4–5 junction to include bilateral common/external/internal iliac, presacral, and obturator nodes as per the latest NRG Oncology consensus [49]. Planning target volume (PTV) was generated by the isotropic expansion of both CTV and CTV_L by 5 mm, except for the 3 mm in the posterior direction for the prostate/SVs. Various organs at risk (OARs) including the rectum, bladder, bowel, penis, penile bulb, femoral heads, and cauda equina were contoured by radiation dosimetrists.

**Figure 1:**
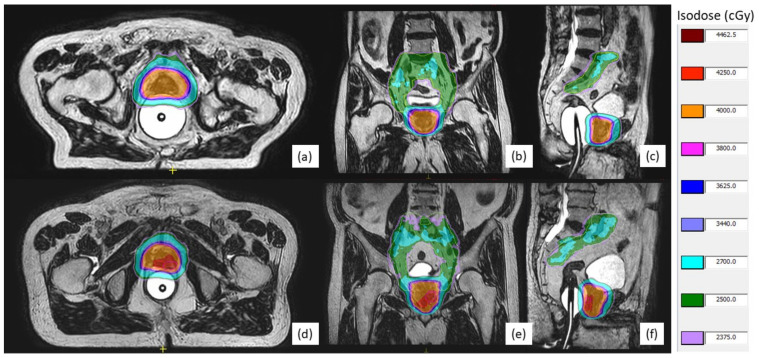
Examples of magnetic resonance-guided stereotactic body radiotherapy plan on high-risk prostate cancer patients without (**a**–**c**) and with (**d**–**f**) dominant intraprostatic lesions (DILs). PTV, PTV_L, and DILs are indicated by orange, green, magenta, and red solid lines, respectively. Isodose levels are also illustrated by different colors.

Intensity-modulated radiation therapy (IMRT) plans with a mean of 15 beams were generated using Monaco v.5.40 (Elekta, Stockholm, Sweden). A dose of 40 Gy was prescribed to CTV, and doses of 25 Gy and 42.5 Gy were prescribed to the pelvic nodal volume and DILs, respectively, in five fractions. The MRgSBRT was delivered twice per week. The planning objectives and dose constraints of targets and OARs are summarized in Supplementary Table S1. Figure 1 shows the examples of plan dose prescription on two HR-PC patients with and without a DIL boost. Androgen deprivation therapy (ADT) was planned in general for a duration of 18 to 36 months using a luteinizing hormone-releasing hormone agonist or antagonist.

### 2.4. Treatment Delivery and Adaptation 

MRgSBRT commenced within 2 weeks after simulation scans for all patients. At each MRgSBRT fraction, patients underwent a daily 3D T2W TSE MRI scan on the 1.5 T MR-LINAC to obtain the on-the-date anatomy information [48]. The same bladder and rectum control as used in the simulation scan was applied. Online plan adaptation was conducted using either the adapt-to-position (ATP) approach, which was prioritized to maximize workflow efficiency, or the adaptive-to-shape (ATS) approach, when substantial anatomical changes were encountered [46,50]. As the pelvic nodal region was closely tied to the bony structures, the alignment of patients with the pelvic bones, such as sacral promontory, pubic symphysis, and right and left ischial spines, was emphasized. The necessity of ATS was usually due to the shape change or the motion of prostate CTV, whilst the pelvic nodal CTV was seldom modified on the basis of accurate alignment with the pelvic bones. If ATS was determined, the attending oncologist adapted the contours of the CTV, rectum, bladder, and bowel via manual contouring and/or deformable registration. Plan re-optimization was subsequently conducted on the basis of the adjusted contours to achieve all planned dosimetric criteria. Optimal target coverage was prioritized while restricting the high dose to OARs. Subsequently, an online patient-specific quality assurance (QA) was performed on the basis of an independent MU calculation approach using RadCalc software (LifeLine Software Inc., Tyler, TX, USA). Another MRI scan was performed for the patient’s position consistency during online plan adaptation, immediately followed by delivery of the radiation dose as planned. Intra-fractional patient movement was not monitored using dynamic MRI during beam-on time. No gating or tracking was performed.

### 2.5. Patient Follow-Up and Outcome Measurements

The follow-ups were scheduled at the completion date of MRgRT, at 1 month, at 3 months, and every 3 months thereafter. Gastrointestinal (GI) and genitourinary (GU) toxicities of MRgSBRT were evaluated as per the Common Terminology Criteria for Adverse Events (CTCAE) scale v5.0. Patient quality of life (QoL) was longitudinally assessed using the Expanded Prostate Cancer Index Composite (EPIC) questionnaire in the domains of urinary function, bowel habits, sexual function, and hormonal function. Prostate-specific antigen (PSA) responses and biochemical recurrences were also assessed.

### 2.6. Statistical Analysis

The follow-up time was calculated between the last follow-up date and the last treatment fraction date. The Shapiro–Wilk test was used to assess distribution normality. Descriptive statistics are presented as means and standard deviations (SD) for normally distributed continuous variables, and medians are presented as ranges for non-normally distributed continuous variables. The Kruskal–Wallis H test or the Mann–Whitney U test was used to compare the longitudinal QoL at different follow-up time points, where appropriate. All statistical analyses were performed using R v1.2 (RStudio, Boston, MA, USA).

## 3. Results

### 3.1. Patient Selection and Baseline Characteristics

Between March 2020 and April 2022, 73 clinically HR-PC patients were eligible for 1.5 T MRgSBRT treatment in our hospital. Among these patients, 31 patients were excluded because nodal or distant metastasis were found using diagnostic multiparametric MRI and/or PSMA PET-CT imaging. Forty-two patients were finally included in this study. Patient and tumor characteristics at the (pretreatment) baseline are summarized in Table 1.

### 3.2. Treatment Delivery and Adaptation

All treatment fractions were successfully delivered for all included patients. In the total 210 (42 × 5) fractions, ATS and ATP were used for online daily treatment adaptation in 21 fractions (10%) for 19 patients and 189 fractions (90%) for 42 patients, respectively. On average, all procedures of patient setup, daily MR imaging, recontouring, plan adaptation, online QA, and dose delivery took 62 min for ATP workflow (range: 49–81 min) and 94 min for ATS workflow (range: 68–131 min). The average beam-on time was 21 min (range: 15–25 min).

### 3.3. Patient Follow-Up and Clinical Outcome

The patient follow-up data were locked at the end of April 2022, ranging from 20 to 609 days (1–20 months) with a median of 251 days.

The clinician-reported GI and GU toxicities are summarized in Table 2. In general, grade ≥1 GU toxicities were more frequently encountered than GI toxicities at both acute and subacute phases. Most patients’ toxicities were resolved during their subsequent follow-ups. Regarding the acute toxicity, the maximum cumulative grade 1 GI and GU toxicities were observed in eight (19.0%) and 34 (81.0%) patients, respectively, while the grade 2 GI and GU toxicities were observed in only one (2.4%) and three (7.1%) patients. As for the subacute toxicity, the cumulative grade 1 GI toxicity slightly increased to 21.4% (nine patients), whereas the grade 1 GU toxicities were less and occurred in 27 patients (64.3%). The grade 2 subacute GI and GU toxicity rates were 2.4%. Neither GI nor GU grade 3 toxicity was found in any patient. The distributions of GI and GU toxicities are listed in Table 3. The most common acute GI toxicities were abdominal pain, proctitis, and rectal pain, each in two patients. In subsequent follow-up, seven rectal hemorrhages (G1: six, G2: one) occurred and resolved with time or medical intervention. Regarding GU toxicity, the most common one was urinary frequency, at both acute (in 31 patients) and subacute (in 25 patients) phases, followed by urinary tract pain in 16 patients at the acute phase and three patients at the subacute phase, respectively. With respect to the patients who experienced grade 2 toxicities, apart from one patient with aforementioned subacute rectal hemorrhage, one patient with acute diarrhea, two patients with urinary frequencies (one acute and one subacute), and two patients with acute urinary tract pain were observed. No patient experienced bloating, fecal incontinence, or nausea during their follow-up period.

**Figure 2:**
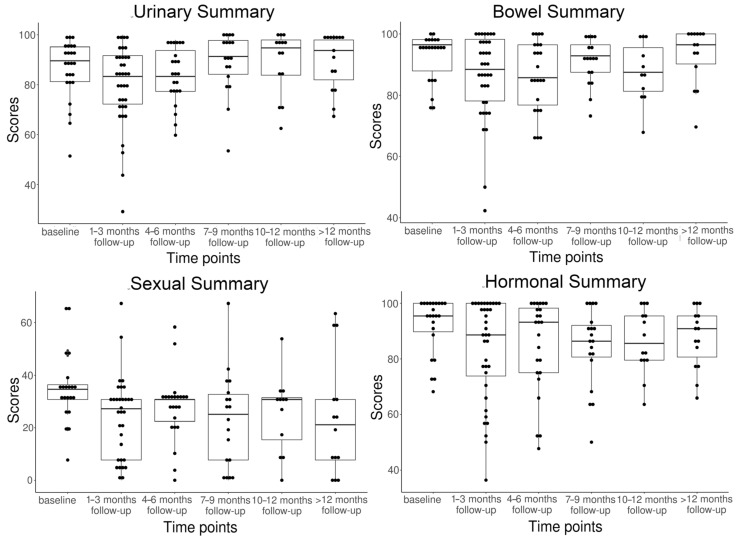
Longitudinal changes in Expanded Prostate Cancer Index Composite (EPIC) domain summary scores during the follow-up period.

Thirty-seven out of 42 patients (overall response rate: 88.1%) gave at least one EPIC questionnaire for QoL measurement, as summarized in Table 4. The sexual score was significantly lower (*p* < 0.05) than other domain summary scores at all follow-ups. All four urinary, bowel, sexual, and hormonal domain summary scores were reduced at the first follow-up timepoint at 1–3 months compared to the baseline scores. Thereafter, the urinary and bowel scores gradually recovered and rose slightly higher than the baseline levels, while the sexual and hormonal scores remained lower at longer follow-ups. All four domain summary scores did not show significant differences (all *p* > 0.05) during the entire follow-up period. The longitudinal changes of EPIC domain summary scores are depicted in Figure 2.

The PSA levels continuously reduced from the baseline (median: 14.2 ng/mL, range: 1.46–866 ng/mL) in almost all patients, and the PSA levels at the last follow-up were as follows: median, 0.05 ng/mL; range, <0.01–41.28 ng/mL. Thirty-nine patients had a PSA level of <0.01–1.0 ng/mL. One patient had a PSA level of 1.0–2.0 ng/mL and another patient had a PSA level of >10.1 ng/mL. All these patients had continuously decreasing PSA levels without up-and-down bouncing. Only one patient developed biochemical recurrence using the Phoenix definition (PSA nadir + 2 ng/mL) at the 18 month follow-up.

## 4. Discussion

To our knowledge, our study is the first to prospectively report the early clinical experience and preliminary outcome of MRgSBRT with concomitant WPRT for localized HR-PC patients. The results in our study demonstrated the feasibility of achieving excellent target and OAR dose–volume constraints by utilizing the exceptional image contrast of inter-fractional daily MR images and the capability of online plan adaptation in the MRgSBRT for localized HR-PC patients. The preliminary clinical outcome highlighted the favorable patient tolerance, low toxicity, and encouraging patient-reported QoLs of HR-PC patients treated by this novel MRgRT for both prostate and pelvic lymphatics concurrently.

With a low α/β value of PC cells, hypofractionated RT or ultra-hypofractionation may result in more substantial radiobiological responsiveness, and the clinical outcome in PC patients may be further enhanced as a consequence [51,52,53]. With the potential radiobiological advantage, as well as the promising outcome with ultra-hypofractionated RT for LR- and IR-PC, SBRT has been actively investigated for HR-PC with or without ADT in recent years [24,27,28,29,30,38,39,40,54,55,56]. In the meantime, conventionally fractionated WPRT in addition to prostate-alone RT has been shown to improve the clinical outcome for HR-PC in the POP-RT study. Nonetheless, among those SBRT studies in HR-PC patients, concomitant WPRT and prostate SBRT were evaluated in only a few of them [27,38,39,40,56].

Heterogeneous results were observed across the few studies that investigated the concomitant use of SBRT and WPRT. The early phase I–II FASTR trial (25 Gy to pelvic nodes and 40 Gy to the prostate in five weekly fractions) reported higher-than-anticipated late toxicities and was terminated before phase II accrual [40]. Murthy et al. [27] enrolled 68 consecutive patients with National Comprehensive Cancer Network (NCCN)-defined high-risk (30%), very-high-risk (16%), and node-positive (54%) PC treated with SBRT (35–37.5 Gy in five fractions, 25 Gy to pelvic nodal regions only for node-positive patients) along with long-term ADT in their analysis. Acute Radiation Therapy Oncology Group (RTOG) grade 2 GI and GU toxicity rates were 12% and 3%, respectively, without any acute grade 3 toxicity. Late grade 3 GI toxicity was 3%. Elective nodal irradiation (ENI) did not increase acute or late GU toxicity. At a median FU of 18 months, 97% patients were alive, and 94% were biochemically relapse-free. The phase1–2 SATURN study [39] delivered 25 Gy to the pelvis and seminal vesicles (SVs) and a simultaneous boost of up to 40 Gy to the prostate in five weekly fractions, along with 12–18 months of ADT in 30 HR-PC patients, followed by 18.5–30.7 months (median FU of 25.7 months) to assess CTCAE toxicity and EPIC QoL. High acute and late toxicities of 46.7% and 52% were observed for grade 2 GU toxicity, along with 3.3% and 32% for grade 2 GI toxicity, respectively, without grade 3 toxicities. Mean (95% confidence interval) EPIC urinary QoL scores were 86.6 (81.9–91.3), 87.1 (81.4–92.6), and 87.9 (80.1–95.7) at baseline, 3 months, and 24 months; bowel scores were 94.1 (91.3–97.0), 93.2 (89.1–97.2), and 92.4 (87.7–97.1), respectively. A subsequent analysis showed that these toxicities were insignificantly different from those reported in an MR-guided high-dose-rate brachytherapy study [56]. In another study by Pinitpatcharalert et al. [38], 23 HR or node-positive PC patients were treated with SBRT (37.5 to 40 Gy to the prostate and seminal vesicles, with concomitant 25 Gy to the pelvic nodes in five fractions) along with 18 months of ADT. The median 19 month (3–48 months) follow-up showed acute grade 1 and grade 2 GI toxicities (CTCAE) of 9.1% and 0%, respectively. Acute GU grade 1, 2, and 3 toxicity rates were 31.8%, 36.4%, and 4.5%, respectively. Late grade 2 GI and GU toxicities were observed to be 9.1% and 27.3%, while grade 3 GI and GU toxicities were observed to be 0% and 4.5% respectively, suggesting the good tolerance of SBRT with pelvic lymph node irradiation.

Recently, a simulation study by Christiansen et al. [47] showed that online adaptive MRgRT might reduce the dose to the surrounding tissues compared to standard RT for HR-PC patients by allowing tighter PTV margins to both the prostate (3 mm, 4 mm, and 5 mm in R-L, S-I, and A-P) and pelvic lymph node (uniform 2 mm) CTVs. Such observed reductions in doses to OARs could translate to reduced risks of acute GI toxicity and late bladder toxicity.

In contrast to the aforementioned SBRT studies that entailed X-ray-based image-guidance, our results showed a more favorable toxicity profile. Despite the relatively short follow-up time, both acute and subacute grade ≥2 GU toxicity rates (3.4%) were remarkably lower than those reported in the previous studies. This reduction might mainly be attributed to the unique capability of online plan adaptation with respect to the prostate primary and the OARs, particularly in the bladder, according to the daily MR images. On the other hand, the extended radiotherapy field in WPRT SBRT imposes risk of radiation damage to the bowels and, hence, higher possibilities of RT-related GI toxicities. In the current study, with MR guidance, the grade 2 GI toxicities only occurred in 3.4% patients. In particular, only one patient experienced grade 2 acute diarrhea. Such debilitating GI toxicities resulting from the excessive radiation to the bowel in WPRT may be further alleviated in MRgSBRT since bowel tissue is better localized with daily fractionation. In line with GI and GU toxicities that recovered over time, the scores for urinary and bowel domain of the EPIC returned to baseline in a similar fashion. On the contrary, the sexual and hormonal scores were lower than the other two domains, as a result of the ongoing ADT. Further follow-up will be continued for evaluation of the long-term PRO in this cohort. Regarding the patient reported QoL outcome, the urinary and bowel EPIC scores were similar to those reported in the phase1–2 SATURN study [39]. One possible factor accounting for the QoL difference might be the different fractionation schemes in the SATURN study, which were five weekly fractions against ours with two fractions per week.

Despite the lack of previous MRgSBRT studies for direct comparisons of clinical outcome, our preliminary clinical outcome seemed to be well aligned with the simulation results by Christiansen et al. [47], although slightly larger PTV margins were applied in our study. Long-term clinical outcome of our study will be presented with further follow-up.

It is also worth noting that dose boosting to DILs was applied for a small fraction of patients in our study. It was shown in the FLAME randomized phase III trial that the addition of a focal boost to the DIL improved biochemical disease-free survival (bDFS) for patients with localized IR- and HR-PC without impacting toxicity and QoL [57], but with conventional fractionation rather than ultra-hypofractionation. The ongoing multicenter phase II hypo-FLAME trial preliminarily showed that simultaneous focal boosting to the DILs, in addition to whole-gland prostate SBRT, had acceptable acute GU and GI toxicity, but without using pelvic ENI [58]. In our study, we demonstrated that concurrent prostate irradiation, DIL boost, and pelvic RT were clinically feasible and achievable by using adaptive MRgSBRT. However, the interplay among these three components is complicated, and their influence on clinical outcome is largely unknown. Our present work could provide a good start in further clinical evaluation of this comprehensive triple-component irradiation strategy.

There were some limitations to this study. This was a mono-institutional nonrandomized study without a control arm. The sample size was relatively small, and the follow-up time was not sufficiently long. Meanwhile, the number of patients substantially decreased in the follow-up. The reported clinical outcomes were considered preliminary, while the long-term outcome results are yet to be observed. The preliminary toxicities and clinical outcomes observed in this study have to be further confirmed by randomized clinical trials in the future. For example, the ongoing phase II SRAM study (NCT03938649) aims to compare the acute toxicities of prostate RT plus WPRT in localized HR-PC patients between SBRT and conventional IMRT. The randomized feasibility SPORT study (NCT03253978) attempts to evaluate SBRT in localized HR-PC with or without elective nodal irradiation. However, neither study is MR-guided and clinical outcomes are yet to be reported. This study only included HR-PC patients without clinically involved lymph nodes (cN1 disease). Actually, a similar plan strategy and dose prescription could also be applicable to locally advanced PC patients with cN1 disease. Furthermore, dose boosting to individual metastatic pelvic nodes can be conducted using adaptive MRgRT [59]. Such a novel approach is currently being investigated in our institution but was not included in this study. It is possible that the PTV margins applied in this study could be further reduced and optimized by investigating the intra-fractional anatomical motions using dynamic MRI during beam-on time. This warrants further study. Lastly, the correlation between clinical outcomes and dose characteristics of adaptive MRgSBRT has yet to be investigated.

## 5. Conclusions

This study, for the first time, prospectively reported the initial clinical experience and preliminary outcome of MRgSBRT with concomitant WPRT to HR-PC patients. The results demonstrated the feasibility of achieving excellent target and OAR dose–volume constraints by online adaptive MRgSBRT utilizing the superior daily MR image contrast. The preliminary toxicity data highlighted the good patient tolerance, low toxicity, and encouraging patient-reported QoLs of HR-PC patients treated by this novel RT paradigm. These findings should be further validated in multicenter clinical trials with larger patient cohorts in the future.

## Figures and Tables

**Table 1:** High-risk prostate cancer (HR-PC) patient characteristics at baseline (*N* = 42).

Characteristics	Number of Patients	Percentage
Age (years)		
Mean ± SD	72.5 ± 6.8	
Range	59–90	
Prostate volume (cc)		
Mean ± SD	53.5 ± 40.6	
PSA level (ng/mL)		
Median	14.2	
Range	1.46–866	
T Stage		
2a	1	2.4%
2b	8	19.0%
2c	9	21.4%
3a	18	42.9%
3b	6	14.3%
Pre-treatment Prostate-Specific Antigen (PSA) (ng/mL)		
<10	12	28.6%
10–20	15	35.7%
>20	15	35.7%
Gleason score		
3 + 3	3	7.1%
3 + 4	7	16.7%
4 + 3	6	14.3%
4 + 4	15	35.7%
4 + 5 or 5 + 4	9	21.4%
5 + 5	2	4.8%
Rectal spacer		
Yes	13	31.0%
No	29	69.0%

**Table 2:** The cumulative incidence of clinician reported gastrointestinal (GI) and genitourinary (GU) toxicities according to Common Terminology Criteria for Adverse Events (CTCAE) 5.0 in the patients (*N* = 42).

Highest CTCAE Grade	Grade 0	Grade 1	Grade 2	Grade 3
	GI Toxicity			
Acute (during MRgSBRT and ≤30 days after MRgSBRT)	78.6% (33/42)	19.0% (8/42)	2.4% (1/42)	0
Subacute (>30 days after MRgSBRT to the last follow-up)	76.2% (32/42)	21.4% (9/42)	2.4% (1/42)	0
	**GU Toxicity**			
Acute (during MRgSBRT and ≤30 days after MRgSBRT)	11.9% (5/42)	81.0% (34/42)	7.1% (3/42)	0
Subacute (>30 days after MRgSBRT to the last follow-up)	33.3% (14/42)	64.3% (27/42)	2.4% (1/42)	0

**Table 3:** The distribution of clinician reported gastrointestinal (GI) and genitourinary (GU) toxicities (grade ≥1) according to Common Terminology Criteria for Adverse Events (CTCAE) 5.0 in the patients (*N* = 42).

	Acute (≤30 Days)			Subacute (>30 Days)		
	Grade 1	Grade 2	Grade 3	Grade 1	Grade 2	Grade 3
**GI Toxicity**						
Abdominal pain	2	0	0	1	0	0
Bloating	0	0	0	0	0	0
Constipation	0	0	0	1	0	0
Diarrhea	1	1	0	1	0	0
Fecal incontinence	0	0	0	0	0	0
Nausea	0	0	0	0	0	0
Proctitis	2	0	0	1	0	0
Rectal hemorrhage	1	0	0	6	1	0
Rectal pain	2	0	0	0	0	0
**GU Toxicity**						
Urinary frequency	31	1	0	25	1	0
Urinary incontinence	4	0	0	1	0	0
Urinary retention	1	0	0	1	0	0
Urinary tract pain	16	2	0	3	0	0
Urinary urgency	3	0	0	3	0	0

**Table 4:** Patient-reported outcome measurement based on the Expanded Prostate Cancer Index Composite (EPIC).

	Time Points						
	Baseline *	1–3 Month Follow-Up	4–6 Month Follow-Up	7–9 Month Follow-Up	10–12 Month Follow-Up	>12 Month Follow-Up	*p-*Value
Patients (*n*)	27	37	25	19	14	15	NA
**Domain Summary Scores (median and range)**							
*Urinary*	89.58 [51.42 100.00]	83.33 [29.17, 100.00]	83.33 [59.75, 97.92]	91.33 [53.50, 100.00]	94.79 [62.50, 100.00]	93.75 [67.33, 100.00]	**0.07**
*Bowel*	96.43 [75.00, 100.00]	88.39 [42.31, 100.00]	85.71 [66.07, 100.00]	92.86 [73.21, 100.00]	87.50 [67.86, 100.00]	96.43 [69.64, 100.00]	**0.193**
*Sexual*	34.62 [7.69, 65.38]	27.23 [0.00, 67.31]	30.77 [0.00, 58.33]	25.08 [0.00, 67.31]	30.77 [0.00, 53.85]	21.15 [0.00, 63.46]	**0.06**
*Hormonal*	95.45 [68.18, 100.00]	88.64 [36.36, 100.00]	93.18 [47.73, 100.00]	86.36 [50.00, 100.00]	85.57 [63.64, 100.00]	90.91 [65.91, 100.00]	**0.40**

* Nonparametric Kruskal–Wallis test was used.

## Data Availability

The data presented in this study are available on request from the corresponding author. The data are not publicly available to comply with our hospital policy on patient privacy protection.

## Supplementary Materials

The following supporting information can be downloaded at: https://www.mdpi.com/article/10.3390/cancers14143484/s1, Table S1: Planning objectives and dose constraints for the organs-at-risk (OARs).
