# Supplementary material for: A Prospective Study of Stereotactic Body Radiotherapy (SBRT) with Concomitant Whole-Pelvic Radiotherapy (WPRT) for High-Risk Localized Prostate Cancer Patients Using 1.5 Tesla Magnetic Resonance Guidance: The Preliminary Clinical Outcome
Source: Cancers (Basel). 2022 Jul 18;14(14):3484. doi: 10.3390/cancers14143484 (PMC9321843; doi:10.3390/cancers14143484)
Supplement: Supplementary file 1 [file cancers-14-03484-s001.zip › cancers-1789906-supplementary.pdf]

Table S1. Planning objectives and dose constraints for the organs-at-risk (OARs).

**Structure**	**Planning objectives**
PTV (prostate)	V42Gy <1cc
	V40Gy > 95%
GTV_Boost (DIL)	V44.625Gy < 1cc
	V42.5Gy > 95%
PTV_L (pelvic lymphatics)	V27Gy<20%
	V25Gy >95%
	OAR dose constraints
Rectum	V41.6Gy < 1cc
	V38Gy < 3cc
	V36Gy < 10%
	V32Gy < 20%
	V20Gy < 50%
Bowel Space	V25Gy < 195cc
	V12.5Gy < 830cc
Bladder	V41.6Gy < 1cc
	V37Gy < 5cc
	V36Gy < 10%
	V20Gy < 50%
Femoral Head	V20Gy < 10cc
	Dmax < 30Gy
Penile Bulb	D2% < 28.5Gy
	V20Gy < 3cc
	Dmax < 40Gy
	Dmean < 16Gy

PTV, whole prostate with 5mm (3mm posteriorly) margin; GTV_Boost, MRI-visible dominant intraprostatic lesions (DILs) with 3-5mm margin; PTV_L, pelvic lymphatics (CTV_L) defined by NRG consensus with 5mm margin.
